# Natural seedling recruitment and regeneration in deforested and sand‐filled Mangrove forest at Eagle Island, Niger Delta, Nigeria

**DOI:** 10.1002/ece3.7262

**Published:** 2021-02-25

**Authors:** Aroloye O. Numbere

**Affiliations:** ^1^ Department of Animal and Environmental Biology University of Port Harcourt Choba Nigeria

**Keywords:** heavy metals, mangroves, nypa palm, *Rhizophora*, sand mining, species diversity

## Abstract

Seed recruitment is a major driver of mangrove restoration globally. It is hypothesized that soil condition and channel hydrology can accelerate seedling recruitment and regeneration after a major disturbance. Species abundance, diversity indices, microbial and chemical concentrations in sand‐filled mangrove forest was studied. Eight plots measuring 487.77 m^2^ each were established with ten transects in each plot in a random block design to investigate the effect of soil conditions on seedling growth. A total of 1,886 seedlings were counted. Seedling abundance was significantly different between red (*Rizophora racemosa*), white (*Laguncularia racemosa*), and black (*Avicennia germinans*) mangroves, and nypa palm (*nypa fruticans*). The most dominant species was black mangrove, and the least dominant species was nypa palm. Muddy soils had the most abundant species (*n* = 994) followed by sandy (*n* = 457) and semi‐muddy (435) soils. Furthermore, sandy soils had the highest species diversity (*H* = 0.896) followed by semi‐muddy (*H* = 0.876) and muddy (*H* = 0.583) soils. The soil metal concentration has no correlation with seed abundance and occur in the order Iron > Nitrate > Copper > Cadmium. Soil with high species diversity had high soil microbial population; however, seedling abundance was correlated with soil nutrients and not heavy metals. Small seeds are easily recruited while good soil condition plus existing hydrological connection facilitated natural seedling regeneration in the disturbed mangrove forest.

## Graphical abstract

This study reveals that natural seedling recruitment and regeneration is facilitated by soil chemicals, seed size, and hydrological channel. Seedling growth is accelerated by soil type, nutrients, and microbial activities.

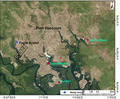

## 1 INTRODUCTION

Natural seedling regeneration involves seed recruitment without any human intervention. For mangroves and coastal ecosystems, it is the growth of forest through tidally assisted natural seedling recruitment without deliberate planting (Teutli‐Hernández et al., 2019). Natural regeneration of forest does not necessarily require site preparation but can occur because of stochastic events that bring about the sudden emergence of natural ecosystem in an area after a major disturbance (Barbier, 2006). Therefore, natural regeneration practice depends on the ecology, phenology, and environmental requirements of tree growth (Jimenez et al., 1985; Kairo et al., 2008). These requirements include the presence of a sufficient number of parent trees that can produce enough viable seeds (Aide et al., 2000; Mckee et al., 2007). Abiotic factors such as optimum sunshine, interconnecting hydrology, and soil chemicals play key roles in the productivity of new forest (Vovides et al., 2011). Mangroves are known to regenerate naturally after earthquake, tsunami, flood (Roy & Krishnan, 2005; Sherman et al., 2000), and oil pollution (Field, 1999). Seedlings can spring up naturally through the aid of tidal current after deforestation caused by oil and gas exploration, sand mining, dredging, and urbanization (Numbere, 2018a).

Deforestation is detrimental to mangroves forest all over the world (Cameron et al., 2019; Krauss et al., 2014). For instance, in the Niger Delta, it has led to the decimation of over 60% of mangrove forest (Numbere, 2018a). Deforestation is the reckless felling of trees to establish projects, which destabilize the natural equilibrium of the ecosystem leading to the loss of numerous ecosystem services. Sand mining activity, for instance, is a prominent business embarked on in the Niger Delta, and is a process by which humans scoop or pump out sharp sand by manual or mechanical means from the bottom of the sea unto land. The evacuated sand is then sold to buyers who use it for construction work (e.g., building projects) and for the filling of manholes (Blanchard & Prado, 1995). Sand mining is a lucrative business in the Niger Delta because 90% of houses are built with blocks, which are molded from sand and concrete. The problem of sand mining is that it impacts the environment negatively when the giant wheels of the bulldozers and swamp buggies destroy the coastal soil and plunders the trees in the proposed site. In addition, trees cut from the site are dumped on nearby fringe forest leading to their suffocation and death. Next, the abandoned dredged materials disfigure the coastlines and acidify the adjoining water body when metal‐laden sediments release harmful chemicals into the water resulting in the death of fishes and other aquatic organisms. Increased sand mining activities in the Niger Delta had converted some mangrove forests into bare land or grassy path. Furthermore, sand dredging activity buries many plant and animal communities (e.g., fiddler crabs, periwinkle, fishes, seedlings, etc.) as well as benthic organisms leading to loss of numerous biodiversity.

The goal of this study, therefore, is to determine the mangrove species abundance and diversity, metal composition, and microbial population in the sand‐filled area. Mangrove and nypa palm seedlings were selected because they are the most dominant species in the study area. The following questions were, thus, addressed: (1) Are there differences in plant species abundance and diversity within plots that have different soil types? If so which part of the plot will have higher species diversity, that is, the channel opening that is muddy or back end that is sandy? (2) Are there differences in chemical and microbial properties of the soil after deforestation and sand filling of the site? (3) Are there correlations between plant species abundance and heavy metal and nutrient concentrations?

## 2 MATERIALS AND METHODS

### 2.1 Description of study area

**FIGURE 1:**
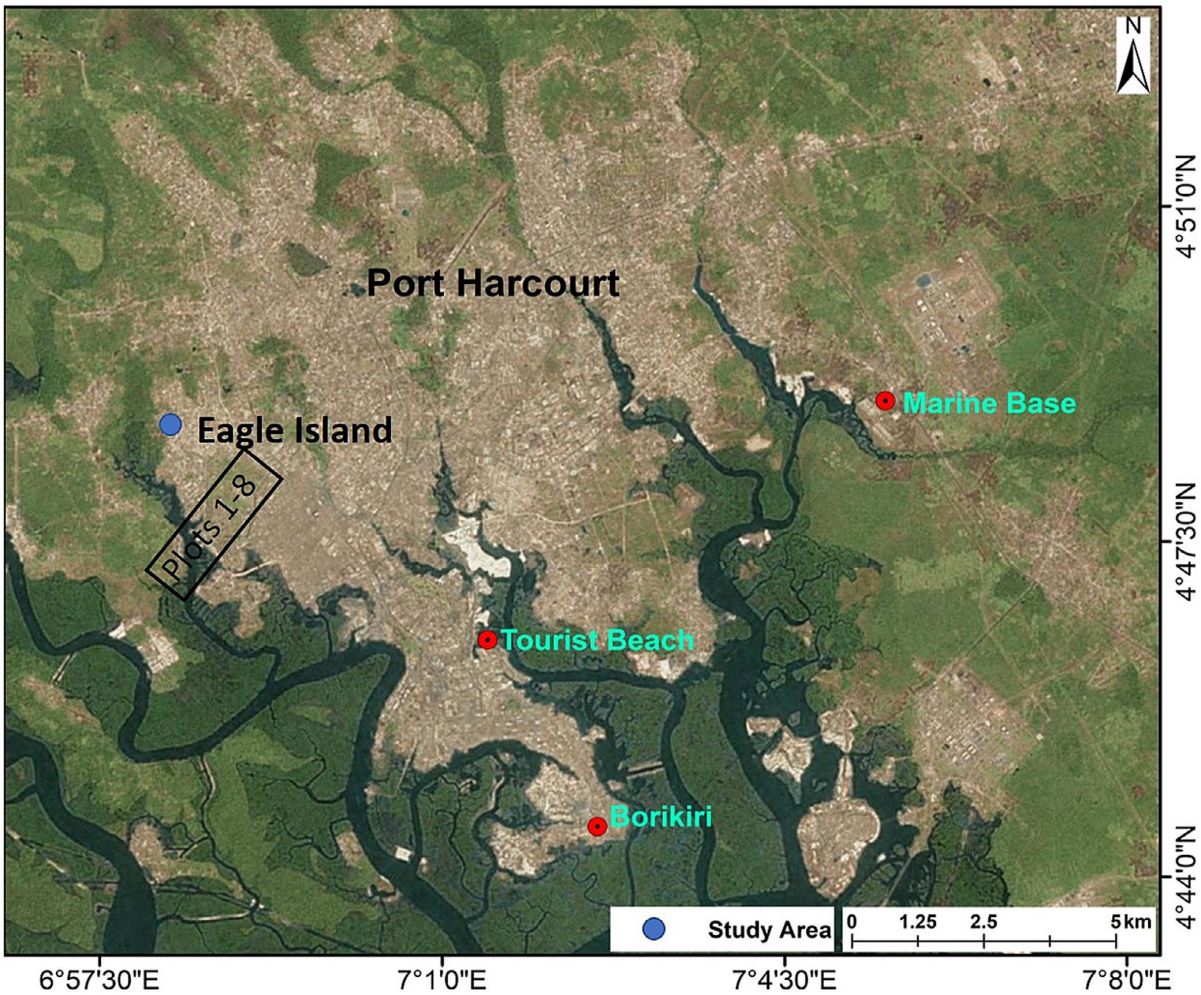
Map of study area at Eagle Island, Niger Delta, Nigeria

The research was conducted in a section of a deforested and sand‐filled mangrove forest behind the Rivers State University at Eagle Island (4°47'N and 6°58'E; Figure 1) in the Niger Delta. The location is a dense mangrove forest that was cut and later used for sand mining activity where dredged sand is dumped. The sand mining business was later abandoned after operating for three years; thereafter, the waterfront of the sand mine became a site for marine transport business, where speed boats were used to convey passengers across the river to a neighboring community. The region experiences rainfall in every month of the year with an annual mean of 3,567.4 mm (Gobo, 2001). The mean monthly temperature ranges from 28 to 34°C. The adjoining river is an estuary with a salinity range of 1.45–1.62%. The soil is sandy to muddy (swampy) and grades from white to dark brown in color. The elevation of the land ranges from 12.20 to 13.41 m from the back end to the front channel (Table 1). Some parts of the sand are red in color due to the presence of iron II (Numbere, 2017). The soil pH ranges from 6 to 7. The study plot is 91 m from the main river course. During high tide, water gushes into the sand‐filled area through a man‐made opening by the side embankment of the site, while during low tide, water rushes out from the same point leaving behind leaf litter, seeds, and seedlings of mangrove and nypa palm.

**TABLE 1:** Soil characteristics in different plots at Eagle Island, Niger Delta, Nigeria

Plots	Coordinates	Elevation (m)	Soil types	Total organic content (TOC)	Pore water salinity (%)	Soil compaction (kg/cm^2^)	pH	Temp (°C)
Plot 1	N04°47.317; E006°58.593	12.20	Sandy	1.02 ± 0.01	1.48 ± 0.02	0.35 ± 0.01	6.7 ± 0.1	29.5 ± 0.1
Plot 2	N04°47.317; E006°58.601	12.20	Sandy	1.01 ± 0.01	1.46 ± 0.02	0.32 ± 0.01	7.1 ± 0.1	28.9 ± 0.1
Plot 3	N04°47.322; E006°58.605	12.50	Sandy	1.11 ± 0.01	1.45 ± 0.02	0.30 ± 0.02	6.8 ± 0.2	29.4 ± 0.1
Plot 4	N04°47.323; E006°58.612	12,50	Semi‐muddy	1.23 ± 0.05	1.51 ± 0.03	0.27 ± 0.01	6.9 ± 0.1	29.6 ± 0.1
Plot 5	N04°47.327; E006°58.624	13.41	Semi‐muddy	1.48 ± 0.01	1.57 ± 0.03	0.25 ± 0.03	7.3 ± 0.1	28.8 ± 0.1
Plot 6	N04°47.320; E006°58.623	13.11	Muddy	1.88 ± 0.01	1.55 ± 0.02	0.22 ± 0.02	6.5 ± 0.1	33.8 ± 0.1
Plot 7	N04°47.329; E006°58.629	13.41	Muddy	1.92 ± 0.01	1.62 ± 0.01	0.23 ± 0.01	7.0 ± 0.2	33.8 ± 0.2
Plot 8	N04°47.328; E006°58.632	13.41	Muddy	1.87 ± 0.01	1.62 ± 0.02	0.22 ± 0.01	6.9 ± 0.1	32.5 ± 0.2

### 2.2 Experimental design

**FIGURE 2:**
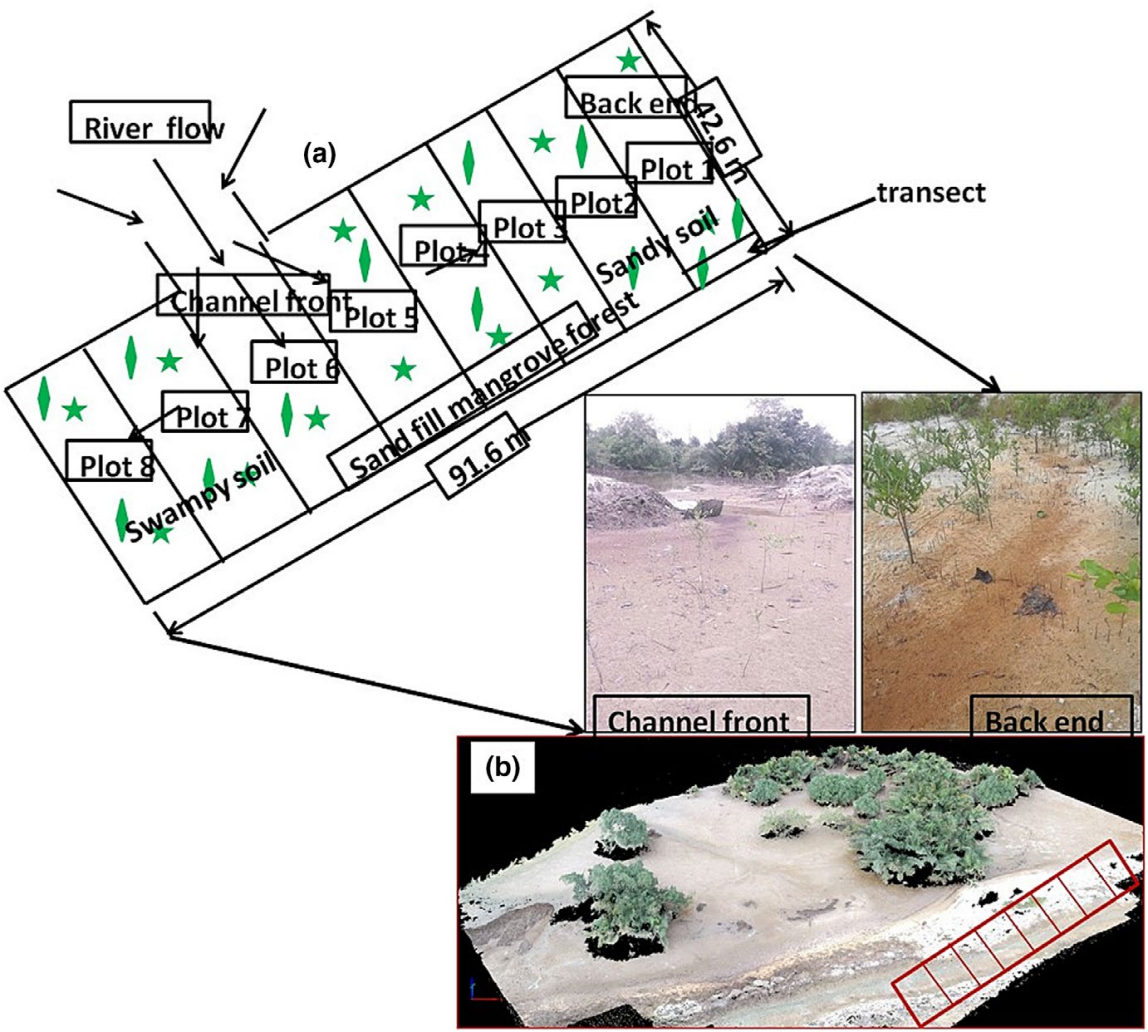
Experimental design of natural seedling recruitment and regeneration study carried out in a (a) 91.6 m × 42.6 m (3,902.16 m^2^) plot at Eagle Island, Niger Delta. The picture indicates the Channel front where river water flows into the sand‐filled area and the back end zone with red iron contamination during low tide. It shows seedlings of different heights (0.3 m–1.22 m) growing naturally; (b) Aerial view of study plot captured by DJI Spark drone at Eagle Island, Niger Delta. Red rectangle represents the study area

The study used a random block design in an area measuring 91.6 m × 42.6 m (3,902.16 m^2^), which was further subdivided into eight plots (habitat types) (Figures 1a and 2a). Within each plot, there are ten (*n* = 10) transects making a total of 80 sampling points. Eight plots measuring 42.6 × 11.45 m each were delineated using a standard tape measure at an accuracy of 0.1 m. Two key areas of the plots are the back end (plot 1) and the channel front (plot 6), which grades from sand to mud, respectively. The sandy soil is made up of 90% sand; the semi‐muddy soil is made up of a combination of sand and mud (i.e., 30% clay and 50% sand); and the muddy soil is made up of 90% silt. The soils were identified in situ and classified using soil textural triangle and soil characteristics of western Nigeria (Smyth & Montgomery, 1962). The front channel is muddy and has a man‐made opening that serves as the entry point of the channel for muddy water during high tide while the back end is sandy and forms an enclosure. The eight plots were georeferenced with a Garmin GPS (USA) (Table 1).

### 2.3 Seedling recruitment and regeneration

Seed and seedlings of mangrove and nypa palm species dominate the area because they are freely brought into the deforested sand‐filled area by tidal current from the nearby standing forest. The deposited seeds are covered with sand and mud and begin to grow into various sizes.

### 2.4 Species abundance and diversity index

All seedlings in sight were counted in situ, and the species abundance recorded in a data sheet. The Shannon diversity Index (H) was used to determine the plant species diversity. It is the natural logarithm which considers low and high species diversities based on abundances. High diversity index means high biodiversity while low diversity index means low biodiversity (Shannon & Weiner, 1949).

### 2.5 Soil sample collection

A hand‐held soil augur was used to randomly collect soil samples 5 cm below the surface within each transect (*n* = 10) in each plot. The samples were placed in well‐labeled polyethylene bags and sent to the laboratory for further analysis. The front channel (~5.0 m wide) and back end areas of the study plots were extensively studied in situ to determine the soil color, texture, compositions, and species distribution (Figure 2b).

### 2.6 Physico‐chemical analysis

Nitrate, iron, cadmium, copper, and zinc were determined using the method of Aigberua and Tarawou (2018) where aliquots of 0.25 g of air‐dried sediment samples were weighed into a Teflon inset of a microwave digestion vessel, and 2 ml concentrated (90%) nitric acid (Sigma‐Aldrich, Dorset, UK) was added. The metals were extracted using a microwave accelerated reaction system (MARS Xpress, CEM Corporation, Matthews, North Carolina) at 1,500 W power (100%), ramped to 175°C in 5.5 min, held for 4.5 min, and allowed to cool down for 1 hr. The cool digest solution was filtered through the Whatman 42 filter paper and made up to 100 ml in a volumetric flask by adding deionized water. The detection limit for the metals analyzed was 0.001 mg/L.

Soil pH was determined with a Kelway soil tester while the soil compaction was determined with a pocket penetrometer. Soil temperature was determined with a digital dual‐sensor thermometer to a detection unit of ±1°C. Salinity of the pore water soil was determined with a salinity meter (OAKTON Salt 6 Acorn Series). The salinity meter probe was used to test standing water in dug‐out holes during low tide.

Total organic content (TOC) was determined using Walkley–Black titrimetric method (Table 1). The TOC was used to determine the nutrients in the soil. The TOC was determined because soil organic content influences soil texture and composition, which in turn influences mangrove growth (Alongi, 2009).

### 2.7 Microbial analysis

Total heterotrophic bacteria (THB) and total heterotrophic fungi (THF) were analyzed from soils collected randomly from each plot. Firstly, the laboratory procedure for the THB was determined as follows: Ig of soil sample was weighed into 9 ml sterile diluents (0.85% NaCl) under aseptic condition. It was then shaken vigorously to homogenize and serially diluted. Then, 0.1 ml aliquot of the inoculums was collected using a sterile pipette, inoculated on nutrient agar surface. The inoculums were spread evenly with a sterile rod. Plates were incubated at 37°C for 24 hr. Thereafter, colonies were counted to obtain colony‐forming unit (cfu) value per ml of the soil sample. Distinct colonies were picked and streaked on freshly prepared nutrient agar medium to obtain pure culture after 24 hr incubation at 37°C. The pure culture was gram stained for microscopic examination. Secondly, the laboratory procedure for the THF was determined as follows: Ig of soil sample was weighed into 9 ml sterile diluents (0.85% NaCl) under aseptic condition. It was then shaken vigorously to homogenize and serially diluted. Then, 0.1 ml aliquot of inoculums is inoculated on Potato Dextrose Agar (PDA) acidified with 0.1% lactic acid to inhibit growth of bacteria and allowed for only the growth of fungi. Inoculated plates were incubated at ambient temperature for 3–5 days. Cultural characteristics of isolates were observed and subcultured for purification. Microscopic examination was done using lactophenol cotton blue stain with ×400 magnifications.

### 2.8 Statistical analysis

An analysis of variance (ANOVA) was conducted since there were multiple samples per block (*n* = 80) to test whether there was any significant difference in plant species diversity within plots and soil types following the example of Quinn and Keough (2002). Similarly, an ANOVA was used to determine if there were any significant differences in metal concentration and microbial population between plots. Logarithmic transformation of the data was performed to meet assumptions of normality and homoscedasticity (Logan, 2010). Similarly, a post hoc Tukey's HSD test was done to investigate pair‐wise mean differences between groups. Pearson's product–moment correlation was done to compare whether there was any significant difference between nutrient and heavy metal concentration and plant species abundance. Regression analysis was also done to compare species abundance and siltiness of the soil. All analyses were performed in *R* statistical environment, 3.0.1 (R Development Core Team, 2013).

## 3 RESULTS

### 3.1 Species abundance

**FIGURE 3:**
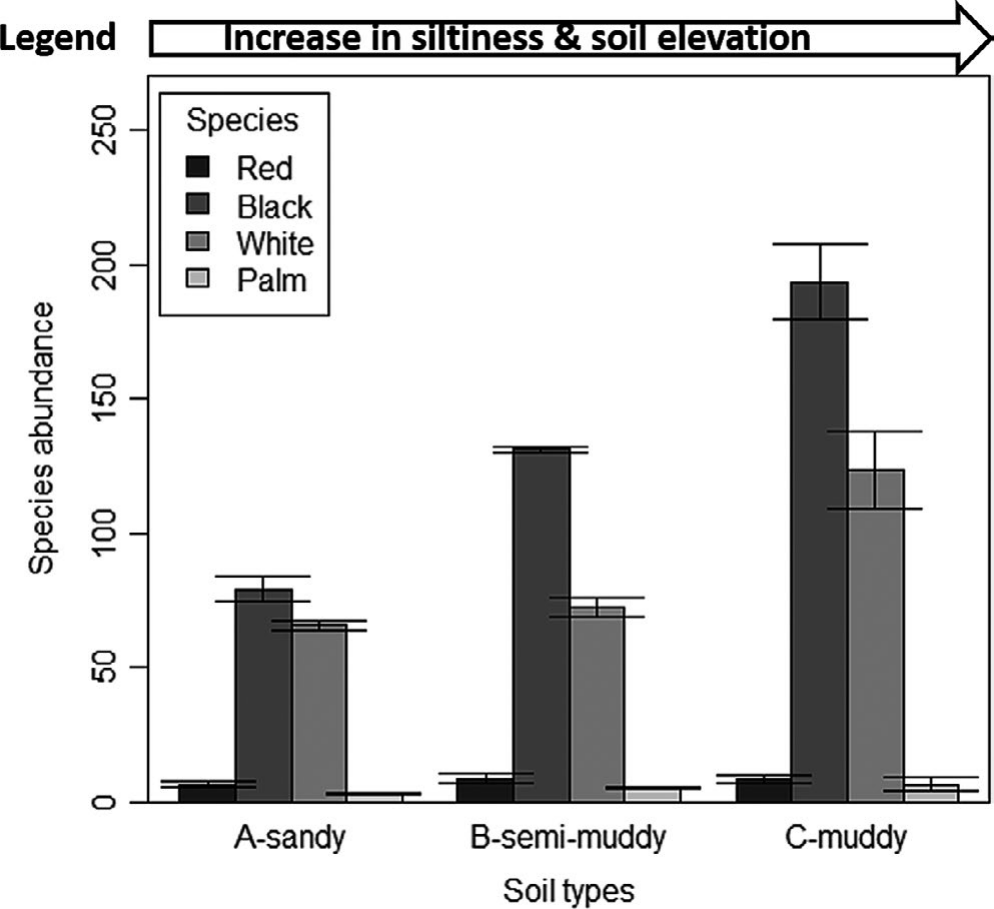
Mean values (±*SE*) of abundance of red (*R. racemosa*), black (*L. racemosa*) and white (*A. germinans*) mangroves and nypa palm (*N. fruitcans*) species in different soil types at Eagle Island, Niger Delta. It shows that front channel area with muddy soil has high species abundance than the back end area with sandy soil. There is also an increase in siltiness (muddy soil condition) from back end to front channel

**FIGURE 4:**
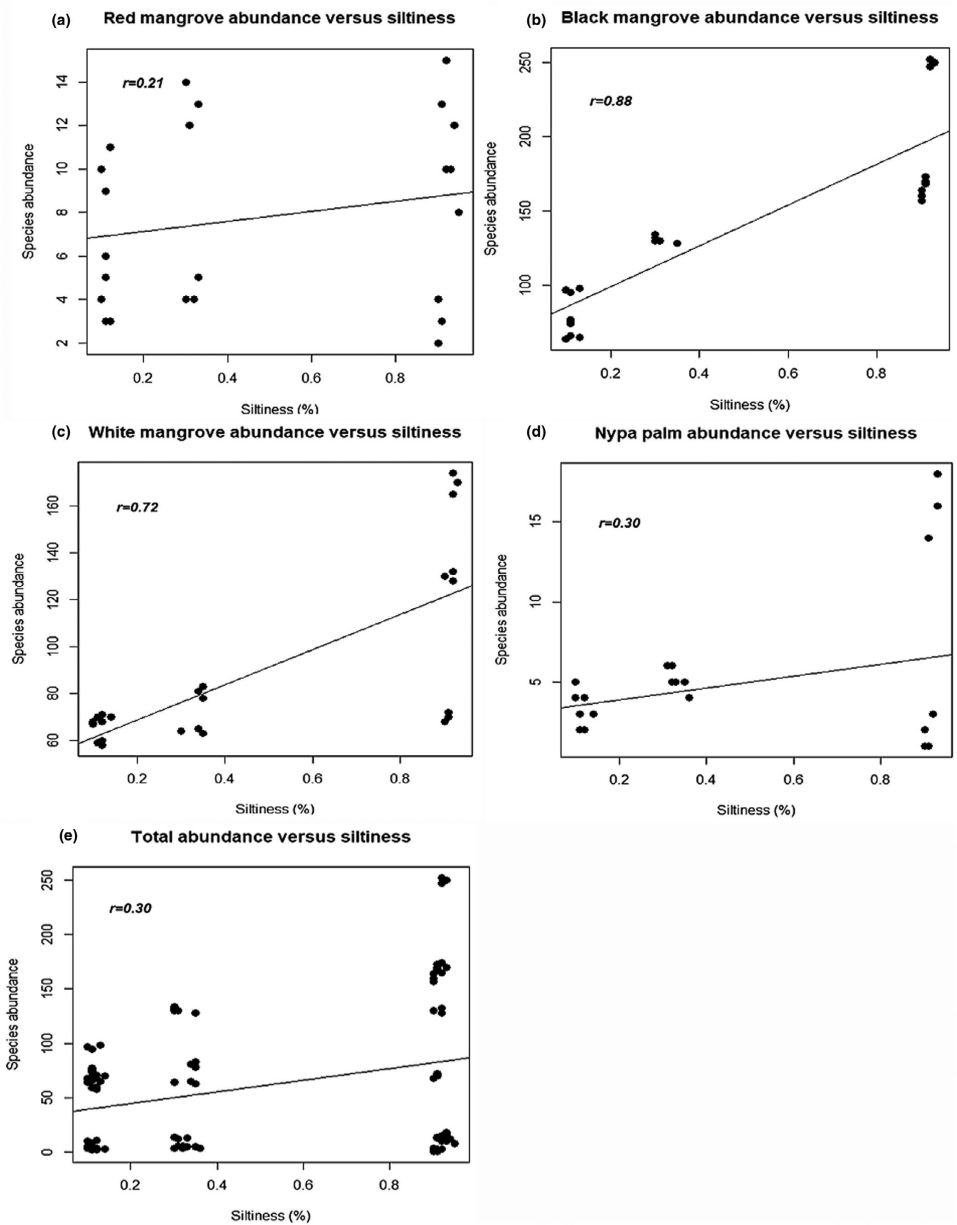
Regression analysis of species abundance versus siltiness (amount of silt present in soil, %) for different mangrove species at Eagle Island, Niger Delta, Nigeria: red (a), black (b), and white (c) mangroves, nypa palm (d), and total abundance (e)

The ANOVA result indicates that there is a significant difference in the abundance of plant species (*F*_3_, _92_ = 82.57, *p* < .0001, Table 2, Figure 3). However, there was no significant difference in the abundance of species between plots (*F*_7, 88_ = 1.95, *p* > .05) (Figure 3). A post hoc Tukey's test showed that black mangroves, nypa palm, and red mangroves differed most significantly at *p* < .05 (Figure 3). Moreover, black mangrove seedlings were the most abundant (*n* = 1,079) followed by white mangrove seedlings (*n* = 709) in all plots. Furthermore, this is shown by high correlation between species abundance and siltiness (amount of silt in sand) for both black (*r = *0.88) and white (*r = *0.72) mangroves (Figure 4). In contrast, nypa palm seedlings were the least abundant (*n* = 35). Plot 8, made of muddy soil, has the most abundant species (*n* = 446) while plot 1, made of sandy soil, has the least abundant species (*n* = 139), which is in line with the Tukey test that showed that the most significant difference in plant species abundance occur between plots 1 and 8 and between plots 2 and 8 at *p* < .05. The front channel has the most abundant species while the back end has the least abundant species (Figure 3).

**TABLE 2:** Species abundance and diversity indices in different plots at Eagle Island, Niger Delta, Nigeria, It shows that the back end zone (Plots 1–4) has higher biodiversity than the front channel zone (Plots 5–8)

Plot	Species	Abundance	Proportion (*P_i_*)	Ln (*P_i_*)	*P_i_* * Ln (*P_i_*)	*H*
Plot 1	*Rhizophora racemosa* (Red)	3	0.0216	−3.835	−0.083	
	*Laguncularia racemosa* (Black)	65	0.4676	−0.76	−0.355	
	*Avicennia germinans* (White)	68	0.4892	−0.715	−0.350	
	*Nypa fruticans* (Nypa palm)	3	0.0216	−3.835	−0.083	
	Total	139				−0.871
Plot 2	*Rhizophora racemosa* (Red)	6	0.0414	−3.185	−0.132	
	*Laguncularia racemosa* (Black)	77	0.5310	−0.633	−0.336	
	*Avicennia germinans* (White)	59	0.4069	−0.899	−0.366	
	*Nypa fruticans* (Nypa palm)	3	0.0207	−3.878	−0.080	
	Total	145				−0.914
Plot 3	*Rhizophora racemosa* (Red)	9	0.0520	−2.957	−0.154	
	*Laguncularia racemosa* (Black)	95	0.5491	−0.6	−0.329	
	*Avicennia germinans* (White)	67	0.3873	−0.949	−0.368	
	*Nypa fruticans* (Nypa palm)	2	0.0116	−4.457	−0.052	
	Total	173				−0.902
Plot 4	*Rhizophora racemosa* (Red)	15	0.0644	−2.743	−0.177	
	*Laguncularia racemosa* (Black)	132	0.5665	−0.568	−0.322	
	*Avicennia germinans* (White)	81	0.3476	−1.057	−0.367	
	*Nypa fruticans* (Nypa palm)	5	0.0215	−3.84	−0.082	
	Total	233				−0.948
Plot 5	*Rhizophora racemosa* (Red)	4	0.0198	−3.922	−0.078	
	*Laguncularia racemosa* (Black)	130	0.6436	−0.441	−0.284	
	*Avicennia germinans* (White)	64	0.3168	−1.15	−0.364	
	*Nypa fruticans* (Nypa palm)	4	0.0198	−3.922	−0.078	
	Total	202				−0.803
Plot 6	*Rhizophora racemosa* (Red)	3	0.0128	−4.358	−0.056	
	*Laguncularia racemosa* (Black)	160	0.6838	−0.38	−0.260	
	*Avicennia germinans* (White)	70	0.2991	1.207	0.361	
	*Nypa fruticans* (Nypa palm)	1	0.0043	−5.449	−0.023	
	Total	234				0.022
Plot 7	*Rhizophora racemosa* (Red)	13	0.0414	−3.185	−0.132	
	*Laguncularia racemosa* (Black)	170	0.5414	−0.614	−0.332	
	*Avicennia germinans* (White)	130	0.4140	−0.882	−0.365	
	*Nypa fruticans* (Nypa palm)	1	0.0032	−5.745	−0.018	
	Total	314				−0.829
Plot 8	*Rhizophora racemosa* (Red)	10	0.0224	−3.799	−0.085	
	*Laguncularia racemosa* (Black)	250	0.5605	−0.579	−0.325	
	*Avicennia germinans* (White)	170	0.3812	−0.964	−0.367	
	*Nypa fruticans* (Nypa palm)	16	0.0359	−3.327	−0.119	
	Total	446				−0.897
	Gross Total	1,886				

### 3.2 Species diversity

The plant species diversity was estimated from the species abundance within each plot. The result (Table 2) indicates that plot 4, made of semi‐muddy soil, has the highest species diversity (*H* = 0.948) followed by plot 2 (*H* = 0.914) and plot 3 (*H* = 0.902), which both are made of sandy soils; whereas, plot 6, made of muddy soil, has the lowest species diversity (*H* = 0.022).

### 3.3 Physico‐chemical analysis

The ANOVA result showed that there is a significant difference between concentration of metals (*F*_3, 60_ = 43.46, *p* < .001, Table 3). But, there was no significant difference in metal concentration between plots (*F*_7, 56_ = 0.69, *p* > .05, Table 3). However, there were higher nitrate and iron concentrations in front channel plots (i.e., 5–8) than in back end plots (1–4) (Table 1). Similarly, there was higher total organic content (TOC) in the front channel plots than the back end plots (Table 1).

**TABLE 3:** Soil metal composition of different study plots at Eagle Island, Niger Delta, Nigeria. Front channel plots (Plots 4–8) have higher metal concentration than back end plots (Plots 1–4)

Metal	Metals (mg/kg)							
	P1	P2	P3	P4	PT5	P6	P7	P8
Nitrate	0.16 ± 0.005	0.13 ± 0.005	0.14 ± 0.005	0.09 ± 0.005	0.15 ± 0.05	0.03 ± 0.001	0.04 ± 0.001	0.03 ± 0.005
Iron	803.0 ± 1.0	349.15 ± 0.85	81.08 ± 1.03	119.47 ± 0.74	991.12 ± 1.18	784.41 ± 3.96	1,096.3 ± 3.70	700.61 ± 1.59
Cadmium	0.001 ± 0.00	0.001 ± 0.00	0.001 ± 0.00	0.001 ± 0.00	0.001 ± 0.00	0.001 ± 0.00	0.001 ± 0.00	0.001 ± 0.00
Copper	90.001 ± 0.01	0.06 ± 0.005	0.52 ± 0.005	0.17 ± 0.005	1.80 ± 0.005	0.001 ± 0.00	1.60 ± 0.00	0.54 ± 0.44

### 3.4 Microbial analysis

**FIGURE 5:**
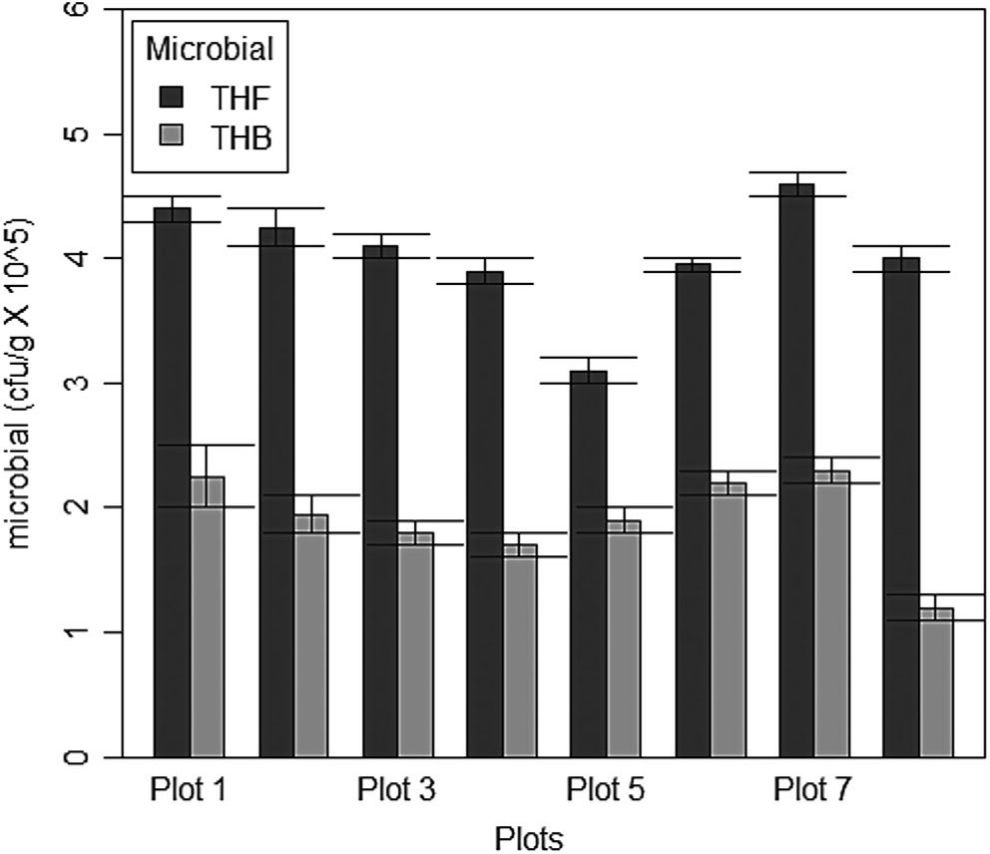
Microbial population in different plots at Eagle Island, Niger Delta

The result indicates that there is significant difference between bacterial and fungal populations (*F*_1, 30_ = 212.7, *p* < .0001, Figure 5), but there was no significant difference in microbial population across plots (*F*_7, 24_ = 0.277, *p* > .05). The post hoc Tukey's test shows that the most significant difference in microbial population is between plots 5 and 7 (Figure 5), which are front channel plots.

### 3.5 Correlation between species abundance and metal concentration

**FIGURE 6:**
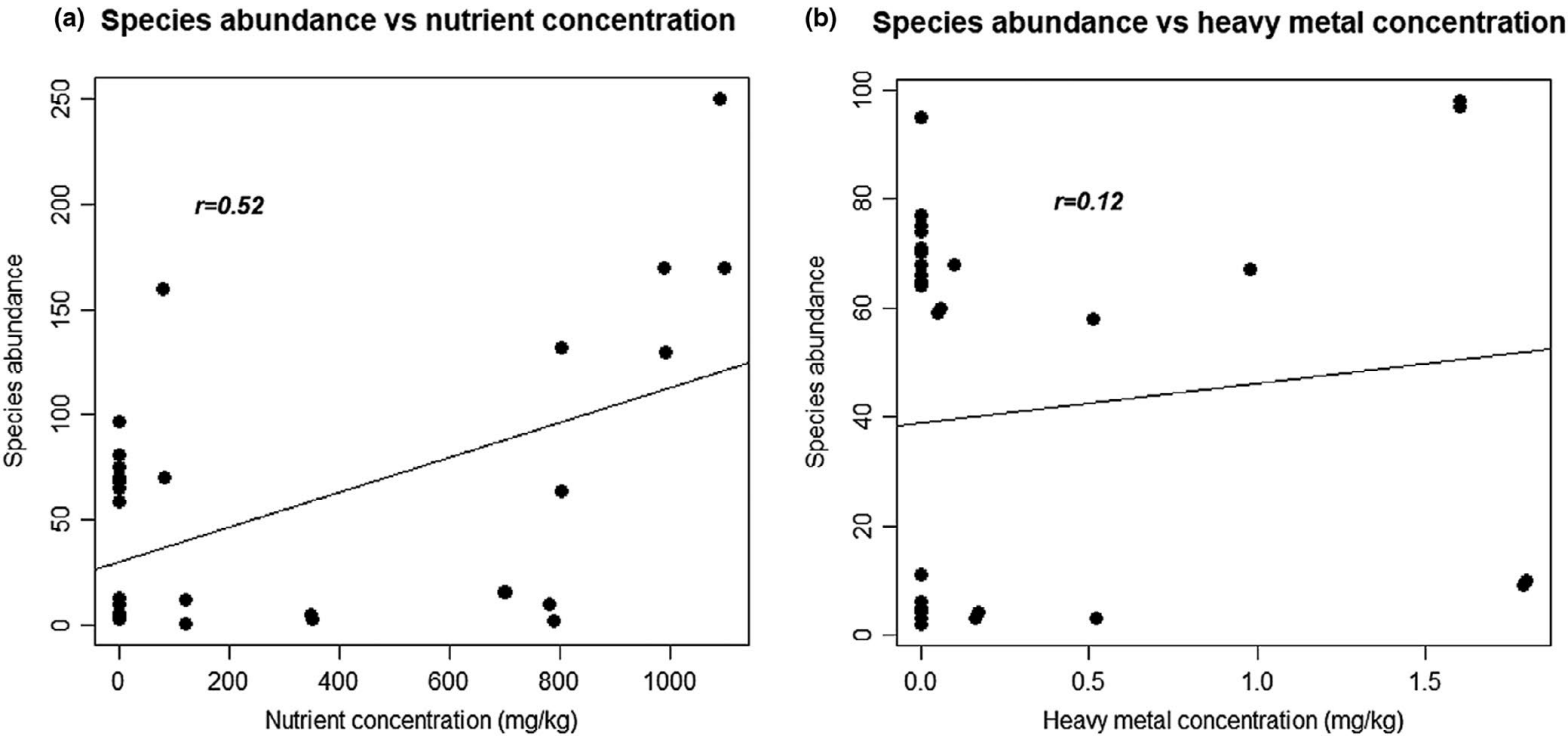
Correlation of species abundance versus nutrient (a), and heavy metal concentrations (b) at Eagle Island, Niger Delta, Nigeria

There was slight correlation between species abundance and nutrient concentration (*t* = 3.3059, *df* = 30, *p*‐value = .002461; cor = 0.5167434), but little or no correlation between species abundance and heavy metal concentration (*t* = 0.65413, *df* = 30, *p*‐value = .518, cor = 0.1185845; Figure 6).

## 4 DISCUSSION

The red mangroves are the most dominant species in the Niger Delta and other regions of the world. But, due to adverse human activities of urbanization, pollution, and sand mining, their population had dropped drastically (Numbere, 2019; Wang et al., 2016). This study, thus, shows that mangroves can regenerate naturally without any human intervention after destruction (Alongi, 2008) if the right environmental conditions are present. For example, after the deforestation of a section of the mangrove forest to establish a sand mine at Eagle Island, it took three years for the seeds to quickly recruit and regrow naturally after the mining venture was abandoned. This study revealed that the natural growth of mangrove and nypa palm seedlings in coastal areas of the Niger Delta and other parts of the world is facilitated by three main ecological factors, firstly, by good soil conditions (Aye & Takeda, 2020) such as the muddiness (siltiness) (Figures 3 and 4) and chemical composition of the soil (Table 3), secondly, by the presence of open hydrological channel and right topography (e.g., elevation and slope of land) (Figure 2) for the inflow and outflow of water and seeds (Lewis et al., 2016), and thirdly, by the size and nature of the seeds.

In this study, soil condition influenced rapid seedling growth, for instance, more seedlings grew naturally in the muddy and nutrient‐rich soils (i.e., front channel) than in the sandy and nutrient‐depleted soils (back end) (Figure 4). Soils in the front channel have slightly high total organic carbon (TOC) (Tables 1) and iron concentration (Table 3) than soils in the back end. Similarly, heavy metals showed no correlation with seedling abundance, but there was increase in iron and copper toward the muddy front end while nitrate and cadmium concentrations were relatively low in all soil types (Table 3). Seedlings brought into a deforested site by tidal current will have poor growth or may die if the soil quality is poor. This quality of the soil depends on the microbial and chemical compositions of the soil. For example, there was a steady drop and later a gradual resurgence in microbial population toward the front end, which is likely caused by tidally accumulated muddy debris or siltiness which accelerates active microbial activity and thus increase in species abundance (3, 4 and 3, 4). Microbial actions are important because microbes decompose litter to fertilize the soil and enhance nutrient cycling. However, the presence of soil microbes is influenced by tannin, an antimicrobial chemical, produced by mangrove leaves. This chemical reduces the bacterial population of the soil and slows decomposition rate (Steinke et al., 1990). Moreover, other nonchemical factors such as tidal height, rainfall, temperature, and salinity influence decomposition rate (Lewis et al., 2016; Woitchik et al., 1997). The study area has low salinity and high temperature which makes it a tropical estuary (Table 1).

Species diversity is also controlled by sandy rather than silty soil conditions because there was a higher species diversity in the sandy soils (plots 1–4) than in the muddy soils (plots 5–8) (Table 2). Previous study had revealed that red mangroves are habitat‐specific and mainly grow in muddy soils as compared with white and black mangroves that grow in sandy soils, and nypa palms that grow in mixed and disturbed soils (Numbere, 2018b). Furthermore, this result is in line with the zonation and successional theories of mangrove forest, which are both influenced by soil chemicals (Lugo, 1980) and crab herbivory (He et al., 2015) with overall impact on mangrove seedling recruitment (Clarke & Kerrigan, 2002). The study by Lugo (1980) further revealed that red mangroves grow in swampy soils in shallow subtidal and lower intertidal region; black mangroves grow in mid intertidal region made up of peat and sand while white mangroves grow in higher intertidal region consisting of sand and mixed soils. Field observations indicate that muddy soils are brought into the study site by tidal currents and distributed longitudinally across the plot with decreasing concentration from the front channel to the back end (Table 2).

Open hydrological channel with right elevation and slope support seedling recruitment too by allowing seed carrying water to flow into the sandfill area (Figure 2a). This factor is what makes the area a unique seed trap as compared with other sand mines in the region, which lacks a hydrological channel or seed trap for seedling recruitment. The channel structure and elevation in the study site facilitated the entry and the deposition of seeds, which eventually begin to grow into seedlings of different sizes. Thus, without the inflow of seed‐laden water, there would be no natural seedling recruitment and regeneration in the area even with the presence of good soil.

The size and nature of the seeds (i.e., lightness and floatability) also play major roles in their dispersal and distribution around the coast. For instance, black and white mangrove seeds, which have average weights of 0.2 g and 2 g, respectively, were more dominant than the red mangrove and nypa palm seeds that have average weights of 26 g and 130 g, respectively. The dominance of the black and white mangroves can be attributed to the lightness of their seeds which facilitated their movement by tidal current into the study area. In contrast, seedlings of red mangrove were less abundant because of the shape and size of their seeds, which is large, long, and heavy, and have poor drag velocity which reduces their rate of dispersal by tidal current. Similarly, nypa palm seedlings were the least dominant but have large numbers of standing parent trees around the study area (Figure 2b) (Numbere, 2018a). Previous study showed that nypa palm seeds were more buoyant than red mangrove seeds because of their round shape and light outer coat which confer high floatability to them (Numbere, 2019). Although, high floatability did not confer high dispersibility in this study. Another field observation revealed that the seeds of the palm are tightly packed to the bunches and hardly fall off from the parent trees as compared with the mangroves species that hang loosely on the parent trees, and easily blown down by wind. Next, root development was also important to recruitment too, because it was observed that seeds germinate when they are covered by soil after they are deposited by tidal current. Red mangrove seeds have advantage because their seeds are viviparous and start to germinate on the parent tree, and later detach and penetrate the soil to grow (Rabinowitz, 1978). However, the major handicap of red mangrove seed is that they hardly germinate when placed horizontally on the soil surface by tidal current. This delays their root development and thus, exposes them to fiddler crab (*Uca tangeri*) consumption and drying from the sun. In contrast, the nypa palm seeds are covered with thick outer coat which protects them from fiddler crab consumption and high temperature. Similarly, poor or damaged seed and seedlings would have little or no growth.

## 5 CONCLUSION

This study shows that mangroves are resilient and can regenerate naturally in disturbed sites if the right environmental conditions are created. Natural seedling recruitment and regeneration can be facilitated, but not limited to only hydrological connections as reported in previous studies, but by seed size, which facilitates seed mobility and dispersal, as well as soil microbial and chemical compositions. These biophysical processes are not present in ex‐situ nurseries where the force of nature drives rapid growth, which may have great implication on restoration of devastated mangrove forest globally and the study of restoration ecology.

## CONFLICT OF INTEREST

The author claims no competing interests.

## AUTHOR CONTRIBUTION

**Aroloye O. Numbere:** Conceptualization (lead); Data curation (lead); Formal analysis (lead); Investigation (lead); Resources (lead); Supervision (lead); Writing‐original draft (lead); Writing‐review & editing (lead).

## Data Availability

Laboratory and species abundance data of this study are publicly available on Dryad at https://doi.org/10.5061/dryad.w6m905qnx.
